# Genome Sequence of *Salarchaeum* sp. Strain JOR-1, an Extremely Halophilic Archaeon from the Dead Sea

**DOI:** 10.1128/MRA.01505-19

**Published:** 2020-01-30

**Authors:** Brian P. Anton, Priya DasSarma, Fabiana L. Martinez, Satyajit L. DasSarma, Mohammad Al Madadha, Richard J. Roberts, Shiladitya DasSarma

**Affiliations:** a New England Biolabs, Ipswich, Massachusetts, USA; b Institute of Marine and Environmental Technology, Department of Microbiology and Immunology, University of Maryland, Baltimore, Baltimore, Maryland, USA; c Instituto de Investigaciones para la Industria Química, Consejo Nacional de Investigaciones Científicas y Técnicas, Universidad Nacional de Salta, Salta, Argentina; d Department of Pathology, Microbiology and Forensic Medicine, School of Medicine, The University of Jordan, Amman, Jordan; Portland State University

## Abstract

An extremely halophilic archaeon, *Salarchaeum* sp. strain JOR-1, was isolated from the east coast of the Dead Sea, Kingdom of Jordan, and sequenced using single-molecule real-time (SMRT) sequencing. The GC-rich 2.5-Mbp genome was composed of a circular chromosome and a megaplasmid. The genome contained 2,633 genes and was incorporated into HaloWeb (https://halo.umbc.edu/).

## ANNOUNCEMENT

Halophilic microbes capable of surviving extreme conditions are of interest for biotechnology and astrobiology (1, 2). To increase our access to genomes of these novel microbes, an extremely halophilic archaeon, *Salarchaeum* sp. strain JOR-1, was isolated from the east coast of the Dead Sea, Kingdom of Jordan, and sequenced. As a result, the number of halophilic archaea from the Dead Sea with a completed genome in GenBank, which includes Halobaculum gomorrense and Haloarcula marismortui, is expanded to three genera (3, 4). These microbes provide the basis for comparative genomic analysis of Haloarchaea isolated from below sea level.

To isolate the new haloarchaeal strain, brine was sampled from 30 cm below the surface of the Dead Sea (31.6998272 N, 35.5811240 E), 415 m below sea level, with a temperature of 34.4°C. The brine was inoculated into *Halorubrum* medium plus trace metals (HLM^+^), and growth was stimulated with shaking at 220 rpm at 37°C under white light illumination in an Innova 43R shaker (5). The enrichment cultures were plated onto HLM^+^ agar plates, and the isolate, an extremely halophilic pigmented halophilic microorganism, was purified by 3 rounds of streaking.

After growing a shaking culture in liquid HLM^+^ to the stationary phase, nucleic acids were extracted using the classic phenol extraction method of Marmur modified for *Haloarchaea* (6), and sequencing was performed using the PacBio Sequel platform (Pacific Biosciences, Menlo Park, CA). Using the manufacturer’s 20-kb library preparation protocol with minor modifications, SMRTbell libraries were prepared from 2 μg genomic DNA sheared to 40 kb with a Megaruptor device (Diagenode, Inc., Danville, NJ). The library was sequenced on a single-molecule real-time (SMRT) cell using the Sequel binding kit version 3.0, 10 h of collection time, and 2 h of preextension time (7). Sequencing subreads were filtered and assembled *de novo* using Hierarchical Genome Assembly Process version 4 (HGAP4) with default parameters. There were 342,641 filtered subreads (mean length, 8,697 bp; mean coverage, 745×) in the preassembly, and 2,782× coverage of the chromosome and 2,237× coverage of plasmid pJOR178 in the final assembly. HGAP4 automatically determined circularity for pJOR178, while the chromosome assembled as a linear contig with 75,204 bp of repeated sequence at the two ends. One copy of the repeat was removed and the contig was circularized; resequencing showed that the single copy was consistent in coverage with the remainder of the molecule.

The Salarchaeum sp. JOR-1 genome sequence was annotated using NCBI’s Prokaryotic Genome Annotation Pipeline (PGAP) build 3190 (8), incorporated into HaloWeb (version r1559404112; https://halo.umbc.edu/), and analyzed using HaloWeb (9) and EMBOSS version 6.6.0.0 (http://www.bioinformatics.nl/cgi-bin/emboss). Default parameters were used for all software unless otherwise noted. The JOR-1 genome was 2,523,371 bp long, with a GC content of 66.2%, with the 2,344,456-bp circular chromosome having 66.3% GC content and the 178,915-bp circular plasmid pJOR178 having 63.8% GC content. Based on its 16S rRNA sequence, the genome was most closely related to that of Salarchaeum japonicum, but it also exhibited similarity to other strains (90 to 99%).

The JOR-1 genome contained 2,633 genes, including a single rRNA operon and 45 tRNA genes. The proteome was highly acidic (10), with a calculated mean pI value of 4.7 based on EMBOSS Pepstats. Nearly all core haloarchaeal orthologous groups (cHOGs) were encoded in the JOR-1 genome (11, 12). It contained multiple copies of genes from several families, which included those encoding the origin recognition complex and general transcription factors, as well as more than 2 dozen transposases (13, 14). A gene cluster for gas vesicle nanoparticles was also present (15). Kinetic data from the sequencing results revealed two major methylated motifs, (m4C)ATG (98.7%) and AGCG(m6A)NC (99.3%), and the gene products recognizing these sequences are named M.SspJOR1I and SspJOR1II, respectively. Cleavage with the m5C-dependent restriction enzyme MspJI (New England Biolabs) revealed an additional motif, GGW(m5C)C, and the gene product recognizing this sequence is named M.SspJOR1III (16).

### Data availability.

The *Salarchaeum* sp. JOR-1 genome sequence has been deposited in GenBank under the accession numbers CP042240 and CP042241, and raw data are available in the NCBI Sequence Read Archive under the accession number SRX6627796.
